# Characterizing mobility patterns of forest goers in southern Lao PDR using GPS loggers

**DOI:** 10.1186/s12936-023-04468-8

**Published:** 2023-02-02

**Authors:** Francois Rerolle, Emily Dantzer, Toula Phimmakong, Andrew Lover, Bouasy Hongvanthong, Rattanaxay Phetsouvanh, John Marshall, Hugh Sturrock, Adam Bennett

**Affiliations:** 1 Malaria Elimination Initiative, The Global Health Group, University of California, San Francisco, CA USA; 2 Department of Epidemiology and Biostatistics, University of California, San Francisco, CA USA; 3 Center for Malariology, Parasitology and Entomology, Ministry of Health, Vientiane, Lao People’s Democratic Republic; 4 Department of Biostatistics and Epidemiology, School of Public Health and Health Sciences, University of Massachusetts, Amherst, MA USA; 5 Divisions of Epidemiology and Biostatistics, School of Public Health, University of California, Berkeley, CA USA; 6 Malaria and Neglected Tropical Diseases, PATH, Seattle, WA USA

## Abstract

**Background:**

In the Greater Mekong Subregion (GMS), forest-going populations are considered high-risk populations for malaria and are increasingly targeted by national control programmes’ elimination efforts. A better understanding of forest-going populations’ mobility patterns and risk associated with specific types of forest-going trips is necessary for countries in the GMS to achieve their objective of eliminating malaria by 2030.

**Methods:**

Between March and November 2018, as part of a focal test and treat intervention (FTAT), 2,904 forest-goers were recruited in southern Lao PDR. A subset of forest-goers carried an “i-Got-U” GPS logger for roughly 2 months, configured to collect GPS coordinates every 15 to 30 min. The utilization distribution (UD) surface around each GPS trajectory was used to extract trips to the forest and forest-fringes. Trips with shared mobility characteristics in terms of duration, timing and forest penetration were identified by a hierarchical clustering algorithm. Then, clusters of trips with increased exposure to dominant malaria vectors in the region were further classified as high-risk. Finally, gradient boosting trees were used to assess which of the forest-goers’ socio-demographic and behavioural characteristics best predicted their likelihood to engage in such high-risk trips.

**Results:**

A total of 122 forest-goers accepted carrying a GPS logger resulting in the collection of 803 trips to the forest or forest-fringes. Six clusters of trips emerged, helping to classify 385 (48%) trips with increased exposure to malaria vectors based on high forest penetration and whether the trip happened overnight. Age, outdoor sleeping structures and number of children were the best predictors of forest-goers’ probability of engaging in high-risk trips. The probability of engaging in high-risk trips was high (~ 33%) in all strata of the forest-going population.

**Conclusion:**

This study characterized the heterogeneity within the mobility patterns of forest-goers and attempted to further segment their role in malaria transmission in southern Lao People’s Democratic Republic (PDR). National control programmes across the region can leverage these results to tailor their interventions and messaging to high-risk populations and accelerate malaria elimination.

**Supplementary Information:**

The online version contains supplementary material available at 10.1186/s12936-023-04468-8.

## Background

Forest-going populations are high-risk populations for malaria in the Greater Mekong Subregion (GMS) [1]. Their activities in the forest and forest-fringes areas – e.g. logging, hunting, farming—increase their risk for malaria [2–12] because of an enhanced exposure to forest mosquitoes—*Anopheles dirus* and *Anopheles minimus* [13, 14]-, the main malaria vectors in the GMS. As malaria declines in the region, national control programmes in the GMS aim to eliminate malaria by 2030 [15, 16] and concentrate their prevention and case detection efforts on forest-goers [17, 18]. Yet, much remains unknown about forest-goers’ mobility patterns and the actual type of trips that they take to the surrounding forest and put them at higher risk.

As described in a recent literature review on malaria and population mobility [19], population movement is frequently mentioned as an obstacle in the fight against malaria. With that said, the authors point out to the scarcity of evidence to support that claim and conclude that it led to an excessive focus on “mobile populations” as a risk group. Malaria programmes are encouraged to go beyond identifying *who* is mobile and instead characterize *how* mobile they are and refocus their efforts on mobility itself. Forest-going populations, often identified as a subgroup within “mobile populations” [11, 17, 18], also refers to a broad range of different risk behaviours in and nearby the forest [3, 20], and the actual mobility patterns behind what “forest-going” means need to be better described.

Micro-scale movement data of forest-goers is essential to understand their role in the transmission of forest malaria in the GMS. Heterogeneity in mobility patterns likely results in diverse exposures to mosquito vectors and heterogeneous risks for malaria. For instance, individuals who travel through the forest for days at a time are likely to play a different role in malaria transmission than individuals who cross the forest to reach their rice field everyday but return home every night. Data on forest-goers’ mobility patterns could also be leveraged to better access these population if geographical or temporal bottlenecks can be identified.

The recent advent of portable global positioning system (GPS) logging devices offers unique opportunities to collect fine-scale mobility data on these populations and characterize their movements in and around the forest. These GPS loggers can provide high resolution data both spatially and temporally and have shown high acceptability in rural settings [21–23]. In previous studies, such devices have successfully been used to assess the importance of individual movement data on the transmission of multiple diseases, such as dengue, schistosomiasis, hookworm or filariasis [24–27] but also malaria [28–30].

In this analysis, fine-scale movement data were collected from forest-goers recruited in a focal test and treat (FTAT) intervention conducted in southern Lao People’s Democratic Republic (PDR). This study is the first to describe the mobility patterns of forest-going populations in the GMS using GPS loggers. A clustering analysis was conducted to characterize the heterogeneity within these mobility patterns and a regression analysis to attempt to further segment forest-going populations in terms of their potential exposure to malaria vectors.

## Methods

### Study area

**Fig. 1:**
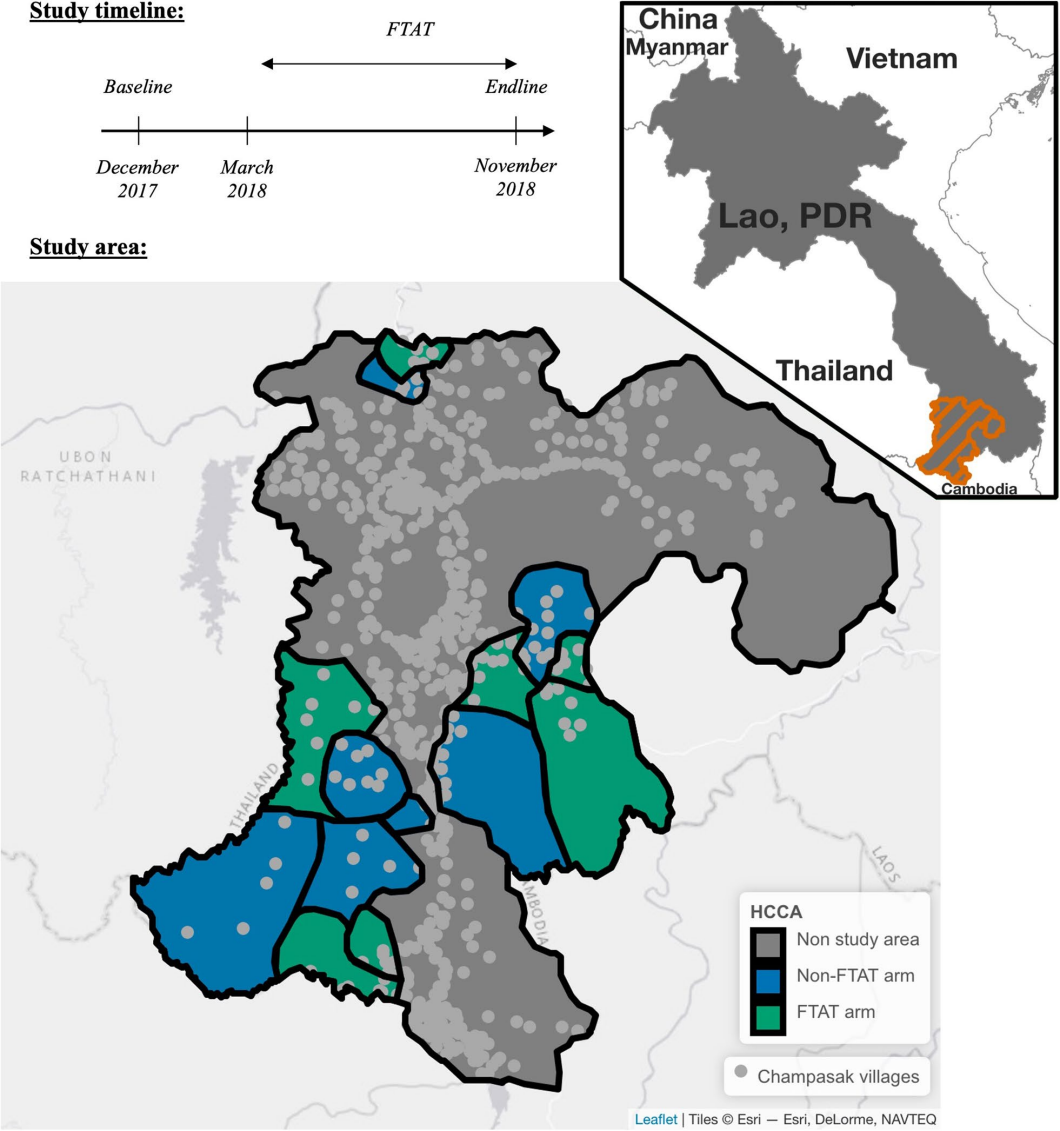
Study timeline and study area. Top left: Study timeline with the FTAT survey conducted continuously between March and November 2018. Bottom: Study area with 7 health center catchment areas (HCCA) out of 14 randomly assigned to FTAT. The study was in southern Lao PDR conducted in the Champasak province that neighbors Cambodia and Thailand (see upper right indent). The map used ESRI imagery available in the leaflet R package

In 2018, a randomized controlled trial was conducted to evaluate active case detection among forest-going populations [31] in southern Lao PDR, where 95% of the country malaria transmission concentrates [32]. The data used in this study was collected among the forest-goers enrolled in the Focal Test-And-Treat (FTAT) arm (Fig. 1), an intervention administered continuously to seven health center catchment areas (HCCA) in Champasak province between March and November.

### Data sources

#### FTAT survey

Fifteen teams of two peer navigators (PNs) were employed to scout forest fringes areas in FTAT HCCAs for individuals presumed to engage in forest-going activities. The eligibility criteria for these targeted “forest-goers” were to be older than 15 and having slept outside of a village on more than one night in the previous month. PNs themselves were recruited, in collaboration with local health authorities, from the local communities of forest-goers and trained to conduct various surveillance activities including blood collection, malaria testing and referrals for treatment. Widely used in other disease areas -especially HIV/AIDS- to sample hard-to-reach groups, the ‘peer-navigators’ technique relies on these community members PNs to actively seek out any persons like themselves for testing, treatment, and linkages to health services [31, 33, 34].

Upon recruitment of forest-goers in FTAT, PNs conducted an epidemiological survey covering the demographic, behavioural, occupational, malaria knowledge and practice domains. To understand the mobility patterns of this population of forest-goers, PNs offered a subset of them, conveniently sampled, to carry a GPS logger that would record GPS coordinates as they carried it.

#### GPS data

In May, 53 GPS loggers (I-gotU 120) were dispatched across the 15 PN teams to be offered to the next enrolled forest-goers and carried for about two months. During that first cycle, loggers were configured to collect GPS coordinates every 30 min and were retrieved in July/August by the PN teams for data downloading. A second cycle of data collection was started in September with 69 GPS loggers configured to collect GPS coordinates every 15 min. Loggers were retrieved in November for data downloading. Recruiting PNs teams also carried GPS loggers, configured to collect GPS coordinates every 30 min over the two cycles.

In order to simplify instructions, the GPS loggers were configured so that they could not be turned off by forest-goers or PNs and the logging intervals selected, 15 to 30 min, afforded an estimated 7 to 12 days of battery life. Loggers could be charged on outlets with regular phone chargers. To avoid battery depletion while on forest trips or off the grid, external charging devices (Verbatim^®^) and two sets of four individual AA lithium batteries were provided to recruited forest-goers. Participants were instructed to carry the GPS loggers at all times, to frequently charge them (at least once a week) and to meet again after two months for GPS loggers’ retrieval. PNs demonstrated all aspects of the GPS loggers’ utilization, including charging, to recruited forest-goers.

#### GPS logger retrieval questionnaire

After roughly two months, PNs met again with forest-goers to collect the GPS loggers in exchange for a $10 monetary incentive. Upon retrieval, a short questionnaire was administered to assess feasibility of using GPS loggers to record mobility patterns of forest-goers. In particular, the survey asked about forest-goers’ charging practices and logger utilization over the two-month study period.

### GPS data processing

#### Data cleaning

The advertised precision of the I-gotU GPS loggers used in this study is 10 m. Yet, the makers warn of possible large errors in the GPS coordinates collected, notably when the logger stay indoor for long periods of time and cannot connect with the satellites. To remove those erroneous GPS points, a filtering algorithm that identifies GPS points unusually far away from both the previous and next GPS points was used. See Additional file 1: S1 for details.

#### Significant locations

**Fig. 2:**
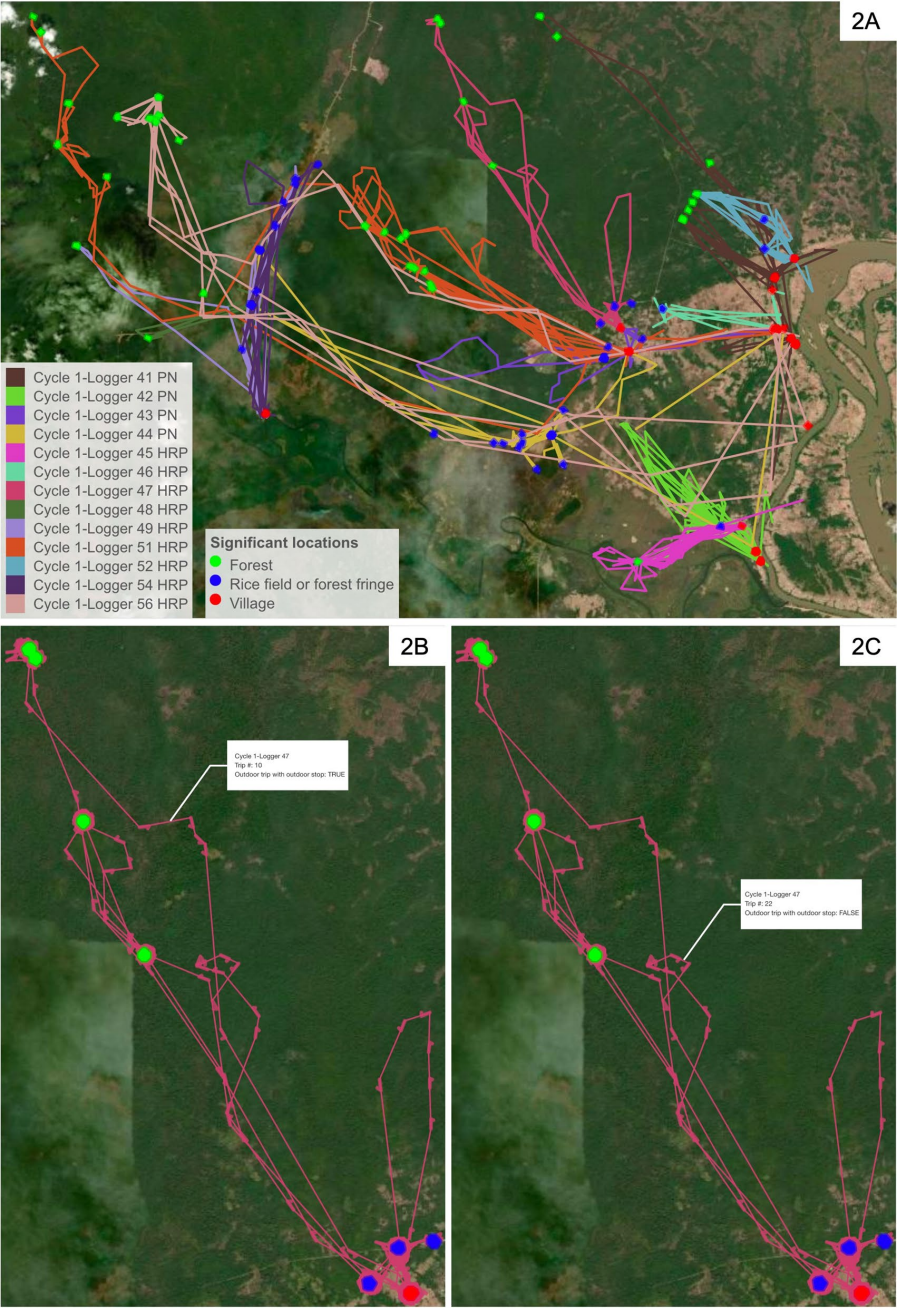
Mobility patterns of forest-goers. Trajectories for GPS loggers collected during the first cycle in Moonlapamok district for PNs and HRPs (High-risk populations), i.e. forest-goers (2A). Figure 2B shows an example of an outdoor trip that stops at an outdoor significant location (trip #10 for GPS logger 47 in 2B). Figure 2C shows an example of an outdoor trip that does not stop at an outdoor significant location (trip #22 for GPS logger 47 in 2C). Significant stop locations are shown as circles, coloured according to their terrain class (Forest vs forest-fringe/rice field vs village). Arrows were added on top of each GPS points in 2B and 2C to represent the travel direction

The data collected by a GPS logger is a time series of GPS points forming a trajectory (Fig. 2a). If several GPS points cluster together, it indicates a location visited frequently or for long periods of time by the HRP carrying the GPS logger (or a location where the GPS logger was left behind). Using a method developed by Barraquand and Benhamou [35] and implemented in the adehabitatLT [36] package in R [37] (version 4.0.5), the residence time spent within a moving 50 m-radius circle window centered on every GPS point of the trajectory was computed. Then, the biased random bridge kernel method [38] implemented in the adehabitatHR [36] R [37] package, was used to estimate the utilization distribution (UD) 30 m per 30 m surface around the trajectory. The UD is a concept widely used in animal movement ecology that measures the utilization of space via the intensity of the GPS points occurrence on the map. A significant location was defined as a 100 m-radius circle centered on a local maximum of the UD surface that contains at least one GPS point of the trajectory with a residence time above 2 h. Simply put, a significant location is a 100 m-radius circle where the GPS logger stayed for more than 2 h at least once along the trajectory.

The resulting 1,068 significant locations were mapped on top of earth terrain layers, using ESRI imagery in the leaflet R package, along with the GPS tracks and manually classified as forest, forest-fringe/rice field or village-based locations by visual inspection. Residence time at village-based significant location as well as self-reported home village by forest-goers in the FTAT questionnaire were additionally used to identify forest goers' home location. Finally, PNs' GPS tracks as well as their self-reported home village were used to identify significant locations that resulted from the study’s activities such as follow-up meetings at PNs' homes. GPS coordinates of forest-goers’ and PNs’ home villages were extracted from a list of geo-referenced villages in the province provided by the national malaria control programme.

#### Outdoor trips

A trip was defined as a series of consecutive GPS points in between two GPS points recorded at the forest-goer's house location. Trips going through an outdoor-based significant location (forest or forest-fringe/rice field) qualified as an outdoor trip (Fig. 2B). Trips where a forest-goer toured the forest for hours but without stopping at a single significant location (Fig. 2C) also need to be classified as outdoor trips. To identify those other outdoor trips (Fig. 2C), a random forest algorithm was used to learn the relationship between the outdoor vs village classification at the 1068 significant locations and the following covariates: number of Open Street Map [39] buildings or places, total 2015 population and average 2018 tree crown cover within 100 m and distance to closest village in the province. Tree crown cover layers came from Hansen [40] and population from WorldPop [41]. The predicting algorithm was the used to classify all other GPS points as outdoor or village-based. Finally, outdoor trips were defined as trips that included either an outdoor-based significant location or a series of consecutive outdoor GPS points adding up to more than two hours. Simply put, an outdoor trip is a trip where the forest-goer spent more than two hours consecutively outdoor. Trips going through a significant location that resulted from the study’s activities were discarded as unrepresentative of the forest-goers’ routine.

### Cluster analysis

For each outdoor trip, the mobility pattern parameters listed in Table 1 were computed. They were selected to characterize forest-goers’ exposure to the dominant malaria vectors in the GMS, *An. dirus* and *An. minimus* [13, 14]*,* all along the trip. Four domains were covered. Two domains, forest surroundings and timing of the trips, pertained directly to the ecology of these mosquitoes, which thrive in a forested environment and bite during nighttime and around twilight and dawn hours (6 pm and 6 am). The two other domains, pace and fragmentation of the trips, reflect the possible organization and habits of those trips and can influence vector control options. For instance, it may be easier to carry bed nets over short distances and frequently visited location along trips may be arranged to offer better mosquito protection.

**Table 1:** Mobility patterns variables

Domain	Forest	Pace	Fragmentation	Timing
Variables	Average 2018 tree crown cover	Duration	Number of stop at significant location	Overnight trip
	Max 2018 tree crown cover	Distance	Proportion of trip spent at significant location	Trip around twilight and/or dawn hours (6 am and/or 6 pm)
	Proportion of trip where 2018 tree crown cover > 50%	Max. speed	Population density	

Mobility patterns variables computed for each of the outdoor trips and used as features in the clustering algorithm (after normalization, standardization, and projection onto the principal components)

Variables in Table 1 were standardized by subtracting the mean and dividing by the standard deviation and right-skewed variables (pace and population density) were log-transformed. Then, principal component analysis was used to project the variables onto the principal components (PC) that captured 95% of the variability in the dataset. Then, hierarchical clustering with the complete distance method was applied on the selected PCs to explore the clustering structure of this dataset of forest-going trips. The hierarchical clustering algorithm starts with one observation per “leaf” (= cluster) and progressively groups similar observations together one at a time until they are all grouped together in a single cluster. An advantage of hierarchical clustering over other clustering algorithms such as k-means is that the number of desired clusters, k, does not need to be set in advance. Instead, the resulting dendogram tree represents the clustering structure for all k from 1 to n, the number of observations. The length of the tree branches quantifies the dissimilarity between the leaves and can be used to assess how many clusters should represent the structure of the data. To help guide the choice of k and its resulting clustering structure, the intra-class correlation coefficient (ICC) for input variables in Table 1 was additionally computed, hence evaluating how many clusters would best capture the variability in the dataset.

Finally, mobility pattern characteristics in Table 1 were summarized for each of the clusters identified and plotted to determine the heterogeneity between the clusters, describe their distributions across the trips, and attempt to classify the type of trips identified in each of the clusters.

### Regression analysis

Outdoor trips including twilights or dawn hours (6am/6 pm) or happening overnight in clusters with high forest penetration were classified as “high-risk” trips given the higher probability of exposure to malaria vectors. Then, gradient boosting trees were used to assess which of the forest-goers’ socio-demographic and behavioural characteristics collected in the FTAT survey best predicted their likelihood to engage in such high-risk trips for malaria. Gradient boosting was selected as one of the most advanced supervised learning algorithms that can accommodate missing values and model non-linearities. Importantly, its implementation in the GPboost [42] R [37] package allows for random effects at forest-goers’ levels to correctly account for the correlation structure with multiple outdoor trips per forest-goers. Automated grid search and fourfold cross validation were used to select the best fitting tuning parameters.

Results are presented using SHAP (SHapley Additive exPlanations) values [43], an innovative tool increasingly used for interpretation of machine learning models. For each of the model’s prediction, SHAP values decompose the contributive importance of each feature and each observation. It enables the ranking of different features in their ability to predict the outcome but also to visualize the adjusted non-linear relationship between the predictors and the outcome.

## Results

### Data description

#### FTAT survey

Over the course of 8 months, 2904 forest-goers were recruited into the FTAT intervention and 122 carried a GPS logger. In terms of acceptability, the field team reported informally that most forest-goers accepted to carry a GPS logger when offered, with only a few refusals. Using answers in the FTAT survey, Table 2 shows how forest-goers recruited in the GPS component of the study differed from those that did not carry a GPS logger. Overall, the two groups were similar although some differences emerged. Forest-goers that carried a GPS logger were older (39.2 vs 36.4 years) and tended to travel in smaller groups (3 vs 4) and for fewer nights (4.1 vs 7.2) than the forest-goers that did not carry a GPS logger. They were also more likely to be male (95% vs 65%), to report forest work as their primary activity (46% vs 28%) and no sleeping structure in the previous night (51% vs 30%) than the forest-goers that did not carry a GPS logger.

**Table 2:** FTAT variables among recruited forest-goers

FTAT variable	Mean among HRP that		
	Carried a GPS logger	Did not carry a GPS logger	p-value
Number of forest-goers in group	3	4.14	< 0.01
Age in years	39.2	36.36	0.01
Number of children	1.79	1.63	0.24
Nights away from home on trip	4.12	7.36	< 0.01
Km away from home	6.63	7.58	0.38
Number of people working/traveling with on trip	2.7	3.62	< 0.01
Ever spent night in forest in rainy season	0.92	0.9	0.62
Ever spent night in forest in dry season	0.94	0.89	0.14
Ethnic minorities	0.07	0.1	0.47
Married	0.87	0.8	0.11
Rice farming is main source of income	0.89	0.92	0.24
Male	0.95	0.65	< 0.01
Education less than primary school	0.43	0.49	0.3
Wood collection is primary reason to visit forest in rainy season	0.32	0.34	0.67
Wood collection is primary reason to visit forest in dry season	0.43	0.48	0.4
Forest work is primary activity this week	0.46	0.28	< 0.01
Motorized main mode of transportation	0.69	0.68	0.81
Relationship to people on trip is family	0.6	0.63	0.55
No sleeping structure last night	0.51	0.3	< 0.01

Comparison between forest-goers that carried a GPS logger and those that did not in terms of their answers to FTAT variables

#### GPS data

**Fig. 3:**
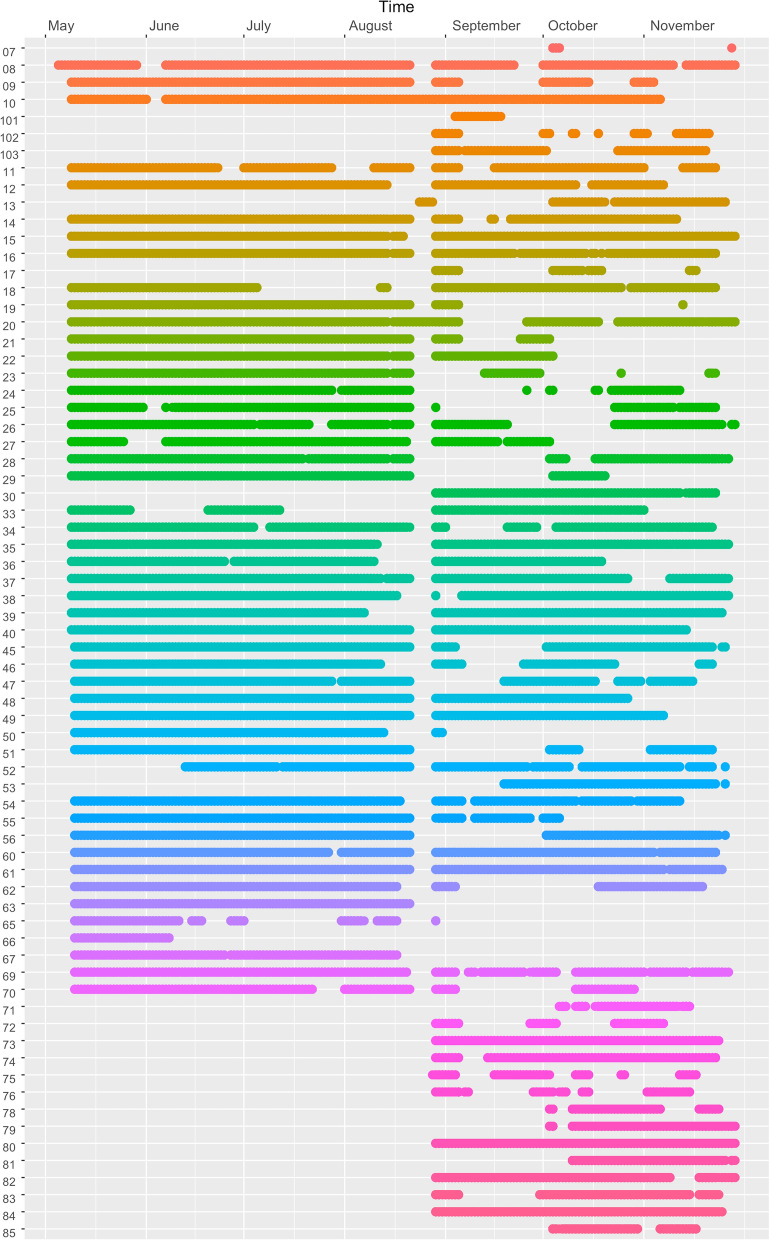
GPS loggers’ time series. Time series plot of when GPS loggers were on and collected GPS coordinates. One row per GPS logger. Gaps indicate times when loggers ran out of battery

Two (1.6%) GPS loggers were not returned and data downloading from 5 (4.2%) others failed, resulting in a total of 472,751 GPS points collected from 115 (94.2%) GPS loggers. Figure 3 shows time series of when GPS coordinates were collected for each of the loggers. The plot shows relatively few gaps, indicating that the forest-goers generally kept their GPS loggers charged. The plot shows the clean demarcation between the two cycles of data collection at the end of August when the loggers were returned to the field team for data download and configuration. For the first cycle, on the left-hand side of the plot, there are almost no data gaps. This motivated us to decrease the logging interval from 30 to 15 min in the second cycle, which resulted in more gaps but provided more battery autonomy to loggers and did not undermine the overall quality of the trip data. Also note that an additional 15 GPS loggers were distributed in the second cycle.

Data visualization exposed a few GPS points obviously logged incorrectly and the filtering algorithm discarded 1973 (0.4%) data points. Most of the time, these errors occurred while the GPS logger was sitting at forest-goers’ house location, most likely beneath some type of roof that disabled connection with the GPS satellites.

Plotting the GPS trajectories also highlighted that forest-goers did not always carry their GPS logger with them. Indeed, some GPS loggers obviously were left at home for weeks at a time. The incentive to give the GPS logger back to the study team after two months may have discouraged forest-goers to take the risk to carry them all the time. Importantly, the study’s instructions insisted primarily on the importance of accurately recording trips to the forest, forest-fringes and rice fields. That is why the analysis focused on outdoor trips rather than on the whole mobility patterns over the two-month study period. In the process, the analysis discarded 95% of the GPS points to focus on the 21,668 (5%) collected along 803 outdoor trips from 96 (79%) forest-goers. The out of the bag error rate of the terrain classification algorithm used to identify outdoor trips was 8.6%.

#### GPS logger retrieval questionnaire

Table 3 summarizes forest-goers’ answers to the retrieval questionnaire conducted when they gave the GPS logger back to the study team. The majority (93.3%) of forest-goers reported to have followed instructions to charge their GPS logger at least once a week. According to the forest-goers, their GPS logger ran out of battery rarely, with 77.5% reporting no battery outages. Surprisingly, 61.7% of forest-goers reported that they shared their GPS logger with another household member, although that happened mostly (80%) for no more than a few days. Only 39.3% of the forest-goers reported carrying their GPS logger every day, which supports the decision to restrict the analysis to outdoor trips only.

**Table 3:** GPS logger self-reported utilization from retrieval questionnaire

Variable	Levels	%
GPS logger ran out of battery	Never	77.5
	1–4 times	15.5
	More than 5 times	7
Charging practice	At least once a week	93.3
	Less than once a week	6.7
Carried GPS	Every day	39.3
	Most of the time	58.1
	Rarely	2.6
Anyone else carried logger	Yes	61.7
Who else	Household member	100
For how long	A few hours	24
	A few days	56
	A few weeks	20

GPS logger self-reported utilization from retrieval questionnaire after forest-goers gave back their GPS logger. N = 120

### Cluster analysis

**Fig. 4:**
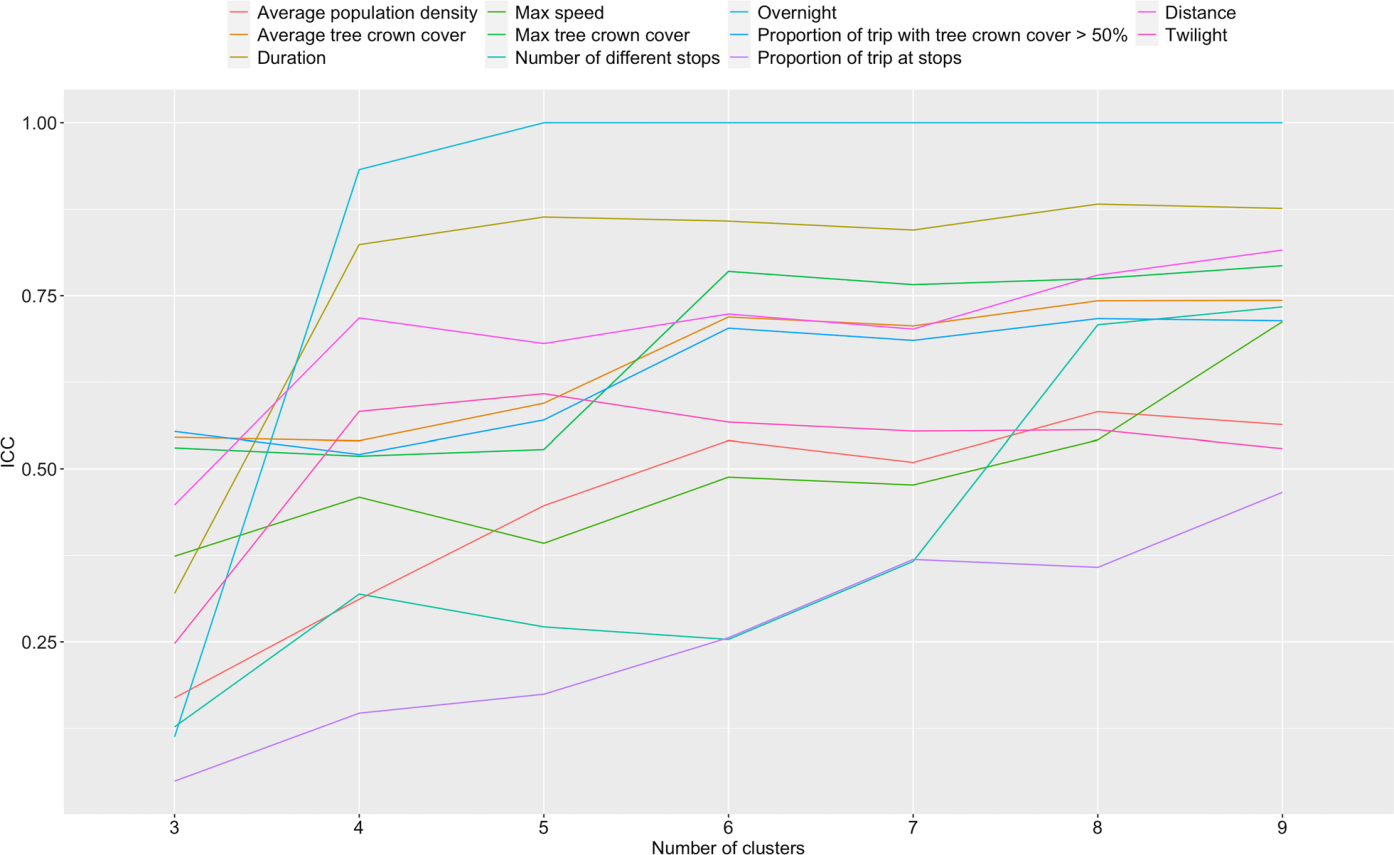
ICC plot. Plot of how the ICC for mobility patterns variables in Table 1 vary with the number of clusters selected. Except for the proportion of trips at stops, the number of different stops and max speed whose ICC continue to improve beyond 9 clusters, for most variables, the ICC increases up to 5 or 6 clusters and then levels off

The first seven PCs accounted for 96% of the variability in the data and were therefore extracted to summarize the outdoor trips data. The dendogram tree (Additional file 2: Fig. S2), resulting from the hierarchical clustering algorithm, is well-balanced and the distribution of large branches suggest cutting down the tree with 6 clusters. In addition, for most of the mobility variables in Table 1, Fig. 4 shows an improvement in the ICC all the way until 5–6 clusters but then levels off. In combination, these plots oriented us to select 6 clusters to summarize the outdoor trips data.

**Fig. 5:**
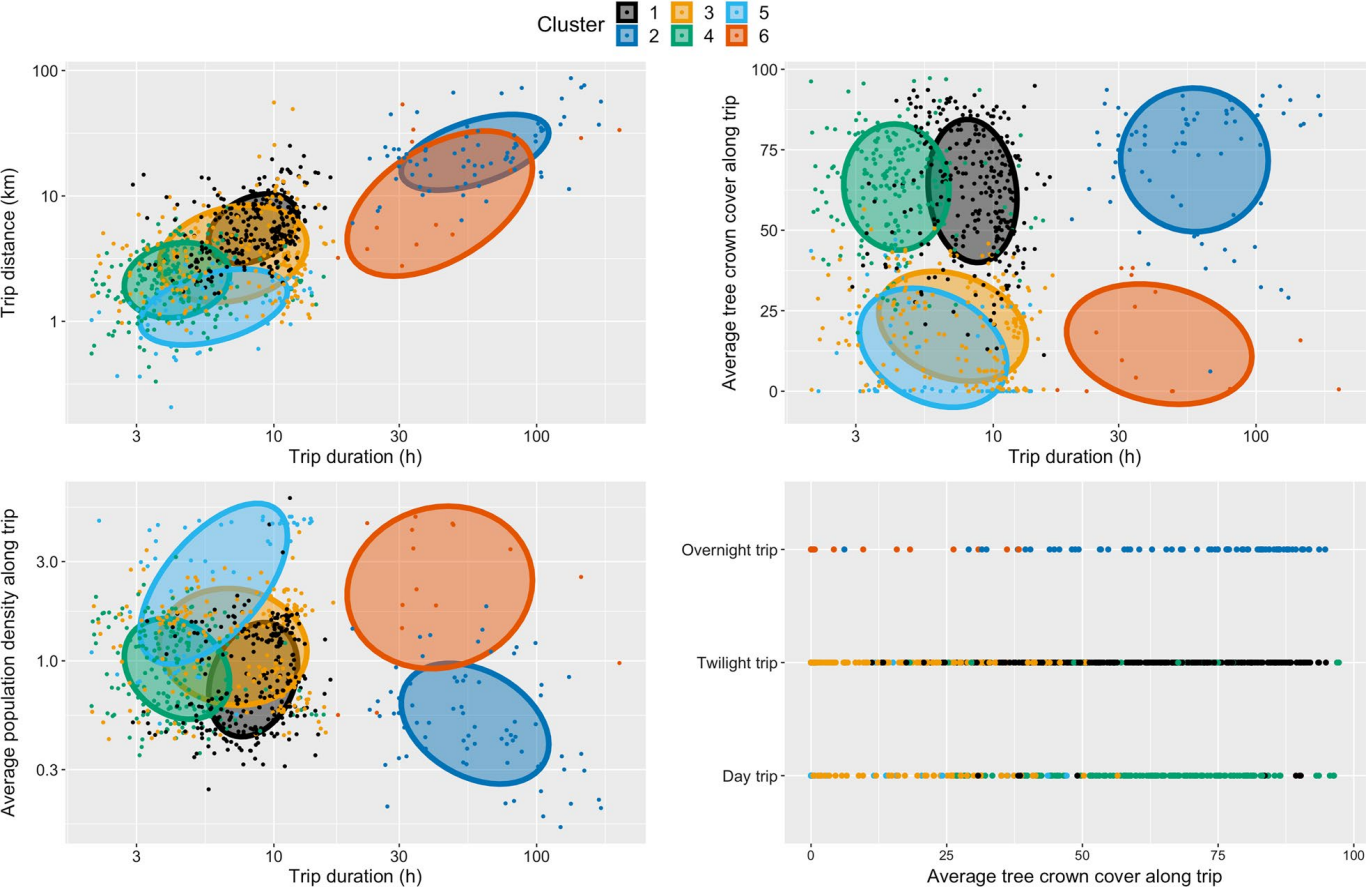
Clustering structure. Bi-plots of the clustering structure in the feature space. Points are coloured by cluster assignment and ellipse capturing 50% of the clusters’ points, assuming bivariate normal distribution, are superimposed. Features represented were selected for their ability to separate the data and highlight the clustering structure of the data

Figure 5 presents biplots of the resulting clustering structure in the feature space. In combination with Table 4, where each of the input mobility variables is summarized by clusters, labeling the 6 types of clusters identified can be attempted. For instance, the darkblue dots of cluster 2 correspond to outdoor trips with high forest penetration and that lasted overnight. As a result, this cluster was labeled “overnight forest trips”. Overall, the recorded forest-goers’ outdoor trips were best differentiated along 3 dimensions (bolded in Table 4): forest penetration, duration/distance and whether the trip happened overnight. Six clusters of outdoor trips emerged: overnight forest trip, overnight non-forest trip, short forest trip, short non-forest trip, day forest trip, day non-forest trip (Table 4).

**Table 4:** Distribution of input mobility patterns parameters for each of the six identified clusters

Cluster	1	2	3	4	5	6
Proposed label	Day forest trips	Overnight forest trips	Day non-forest trips	Short forest trips	Short non-forest trips	Overnight non-forest trips
Count (%)	275 (34%)	75 (9%)	183 (23%)	197 (25%)	58 (7%)	15 9 (2%)
**Percent of overnight trips (%)**	**0**	**100**	**0**	**0**	**0**	**100**
Percent of twilight/ dawn trips (%)	97.1	0.0	71	21.8	46.6	0.0
Mean average tree crown cover along trip [IQR]	62.2 [49.1; 77.5]	71.8 [59.6; 85.9]	20.2 [6.2; 31.3]	63.4 [52.6; 75.2]	13.5 [0; 26.3]	14.6 [0.5; 28.5]
Mean max tree crown cover along trip [IQR]	84.7 [79.3; 93.4]	90 [85.6; 95.1]	38.6 [22.4; 55.6]	81.5 [75.4; 91]	16.4 [0; 27.4]	41.9 [6.4; 78.7]
**Mean proportion of trip with tree crown cover above 50% [IQR]**	**0.7 [0.5; 0.9]**	**0.8 [0.8; 1]**	**0.1 [0; 0.1]**	**0.7 [0.6; 1]**	**0 [0; 0]**	**0 [0; 0]**
**Mean trip duration (h) [IQR]**	**8.8 [6.7; 10.7]**	**67.4 [36.4; 83.5]**	**8 [4.5; 11.2]**	**4.7 [3.3; 5.2]**	**6.8 [3.9; 10.4]**	**55.6 [30.8; 48]**
**Mean trip distance (km) [IQR]**	**6.4 [3.7; 8.1]**	**26.6 [15.8; 31.7]**	**4.9 [1.9; 5.2]**	**2.5 [1.5; 3.2]**	**1.5 [1; 2]**	**15.2 [3.5; 28]**
Mean max speed along trip (kmh) [IQR]	3.6 [2.2; 3.9]	6.2 [3.5; 7.4]	3.3 [1.8; 4.1]	1.8 [1.1; 2.4]	1.2 [0.9; 1.7]	3.9 [1.4; 6.7]
Mean proportion of trip at significant location [IQR]	0.5 [0.2; 0.7]	0.7 [0.7; 0.9]	0.6 [0.2; 0.9]	0.3 [0; 0.6]	0.8 [0.7; 1]	0.8 [0.7; 1]
Mean number of stop at significant location along trip [IQR]	2 [2; 3]	3.1 [2; 3]	1.7 [1; 2]	1.3 [0; 2]	1.7 [2; 2]	2.3 [2; 2.5]
Mean average population density along trip [IQR]	0.9 [0.5; 1.3]	0.6 [0.3; 0.8]	1.3 [0.7; 1.7]	1 [0.6; 1.4]	3 [1.1; 4.6]	2.8 [1.6; 4.4]

**Fig. 6:**
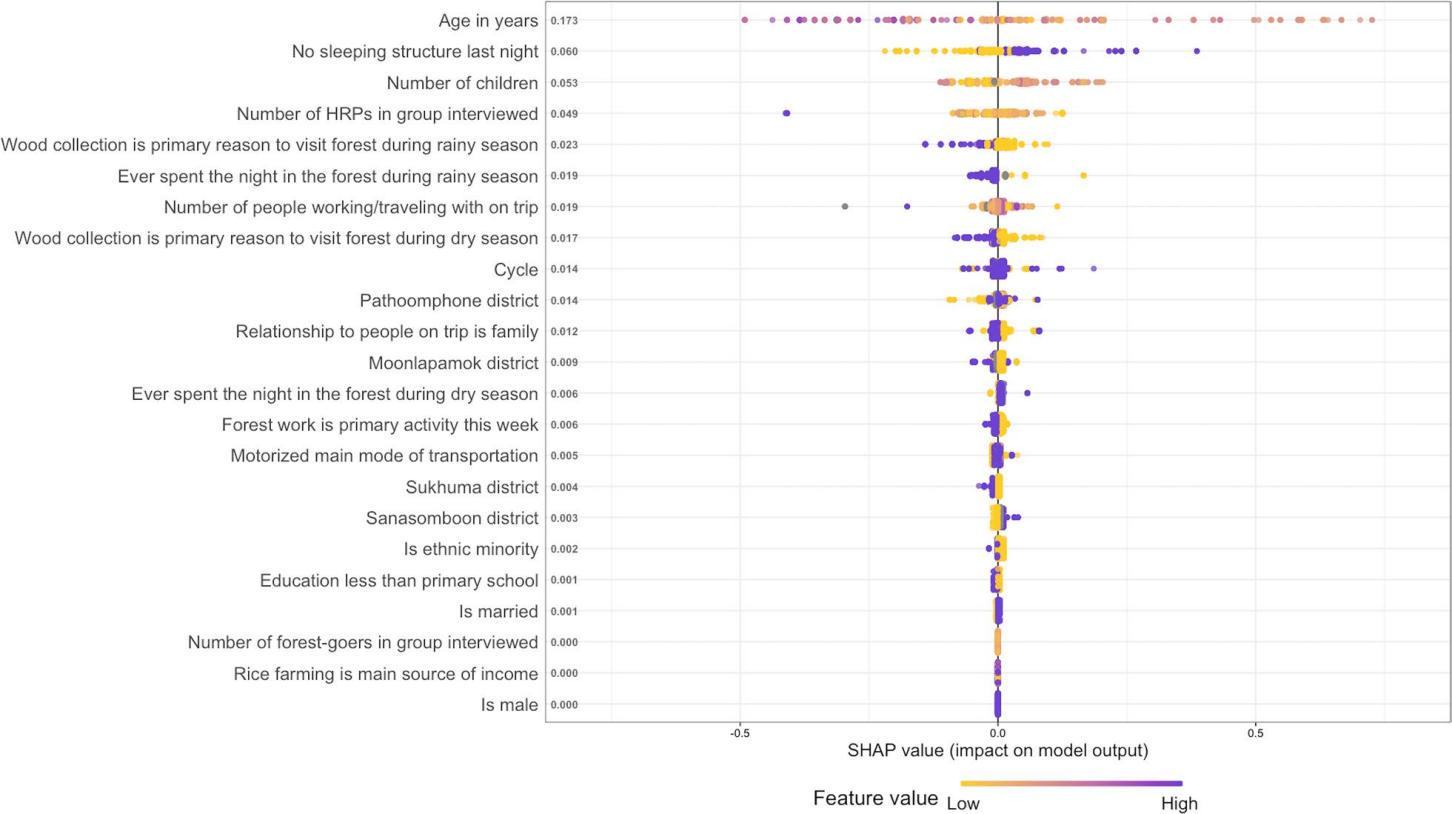
SHAP importance plot. Forest-goers’ individual features are ranked in terms of their ability to predict the likelihood of forest-goers to engage in high-risk trips. For each feature, there is one dot per forest-goers, coloured by the feature value and positioned according to its SHAP value. Larger SHAP values means larger impact on the model predictions. Positive SHAP values result in an increased probability to engage in high-risk trips. The ranking of features is based on the average absolute SHAP value across all forest-goers

Along with Fig. 6, these numbers are suggestive of what the best labels would be for the clusters

Unsurprisingly, trip duration and trip distance were positively correlated while population density and forest cover were negatively associated. Most outdoor trips tend to stop on at least one occasion and forest-goers spend on average between 30 and 80% of their trip time at a stop location. About two thirds (66%) of the outdoor trips collected were classified as forest trips and just over 10% of outdoor trips happened overnight. Overnight trips are also the longest both in duration and distance covered.

### Regression analysis

**Fig. 7:**
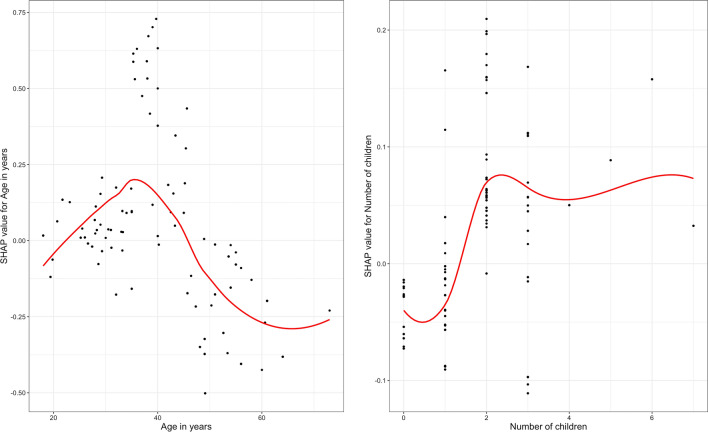
SHAP dependence plot. SHAP dependence plot for the two main continuous predictors of high-risk trips. For each feature, there is one dot per forest-goers. Larger absolute SHAP values means larger impact on the model predictions. Positive SHAP values result in an increased probability to engage in high-risk trips. Super-imposed red lines were modeled using loess with 0.9 span

Overnight forest trips as well as forest trips and short forest trips that happened around twilight and/or dawn hours (6 pm and/or 6 am) further defined 385 (48%) high-risk trips because of their presumed higher exposure to malaria vectors. Figure 6 presents the results from the regression analysis. Individual-level characteristics of the forest-goers collected in the FTAT survey are ranked in terms of their ability to predict forest-goers’ probability to engage in high-risk trips for malaria. Because all the features were collected at the individual level, for each feature, there is one dot per forest-goer, coloured by the feature value. The SHAP value represents the change (additive scale) in the forest-goers’ probability to engage in high-risk trips. The more positive the SHAP values (right side), the higher is their probability to engage in high-risk trips. For instance, forest-goers who reported no sleeping structure the night before their FTAT interview (high feature value, coloured in purple) have positive SHAP values which increase their probabilities of engaging in high-risk trips. On average, forest-goers’ sleeping structure the night before their FTAT interview changed their probability to engage in high-risk trip by 6.1%. For continuous variables, the whole SHAP dependence plots (Fig. 7) can be drawn for more interpretability. For instance, forest-goers aged between 30 and 45 years have high positive SHAP values. Therefore, they tend to have higher probabilities of engaging in high-risk trips than younger and older forest-goers. On average, forest-goers’ age influences their probability to engage in a high-risk trip by 17.4%. For some forest-goers, their middle age increased their probability to engage in high-risk trip by more than 25%.

**Fig. 8:**
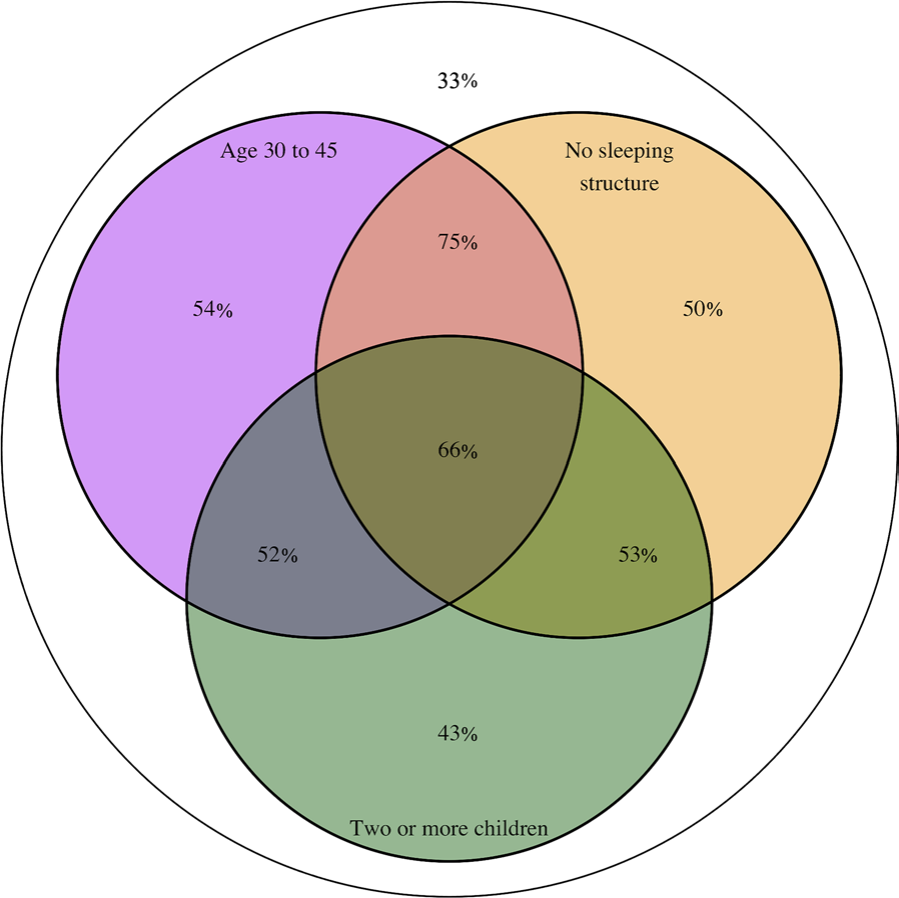
Venn diagram. Venn diagram for the raw probability of engaging in high-risk forest trips among the 8 strata of forest-goers defined by the three main predictors identified in the regression analysis: age between 30 and 45, no sleeping structure the night before the FTAT interview and more than two children. Probabilities are expressed in rounded percent. For instance, the baseline probability of engaging in high-risk trips among forest-goers not aged between 30 and 45 and who reported sleeping in a structure the night before their FTAT interview and who have less than 2 children is 33%

Together, these results have identified age, lack of outdoor sleeping structure and number of children as the best predictors of high-risk outdoor trips for malaria. Specifically, being 30 to 45 years old, using no structure when sleeping outside and having more than two children all increase the probability for a forest-goer to engage in high-risk trips in terms of their exposure to malaria vectors. All the other features impact forest-goers’ probability to engage in high-risk trips by less than 5% on average. As a summary, Fig. 8 presents the probability of engaging in high-risk trips among forest-goers in the 8 strata defined by those three main predictors. These predictors, in combination, increased the probability of engaging in high-risk trips up to 75%. The reference probability of engaging in high-risk trips among forest-goers not aged between 30 and 45 and who reported sleeping in a structure the night before their FTAT interview and who have less than 2 children was 33%. The average probability of engaging in high-risk trips in the seven non-reference strata was 54%, only slightly higher than the unstratified average (48%).

## Discussion

Using GPS loggers to capture fine-scale mobility patterns of 122 forest-goers in southern Lao PDR over two-month periods, data on 803 trips to the forest, forest-fringes or rice fields were extracted. A hierarchical clustering algorithm was used to describe the heterogeneity within these mobility patterns and highlight six major types of outdoor trips. Using gradient boosting trees, forest-goers’ age, lack of outside sleeping structures and number of children were identified as the best predictors of their likelihood to engage in trips at higher risk for malaria, in terms of an increased exposure to mosquito vectors. Together, these three risk factors defined strata of forest-goers with probability as high as 75% and as low as 33% to engage in such high-risk trips.

A key finding from this study is the diversity in forest-goers’ mobility patterns highlighted in the cluster analysis. The 803 outdoor trips collected were highly heterogeneous. Some trips lasted no more than 3 h while others lasted up to a week. Distance covered ranged from 1 to 100 km. Most trips were day trips, with only around 10% happening overnight. The average tree crown cover along the trip ranged from 75% down to around 5%, even for long trips. Six clusters of outdoor trips were identified with major differences in terms of forest penetration, distance covered, duration and whether the trip happened overnight. These differences likely translate into different exposures to the dominant malaria vectors in the GMS, *An. dirus* and *An. minimus*^13,14^, which thrive in a forested environment and bite during the night and around twilight and dawn hours. This heterogeneity in forest-goers’ outdoor trips and exposure to the surrounding mosquito vectors echoes the result from a recent systematic review that focused on qualitative studies on forest-goers in the GMS [20] and called for a finer description of the forest activities that increase malaria risk among forest-goers. Importantly, future survey-based studies about mobility patterns of forest-going populations in the GMS can now leverage the heterogeneity highlighted in this study to tailor their questionnaire and collect representative data at a cheaper expense.

This study attempted to leverage this heterogeneity in mobility patterns to segment the population of forest-goers and identify sub-groups at potentially higher risk for malaria because of their increased likelihood to engage in high-risk trips. This study was able to rank individual level characteristics of forest-goers collected in the FTAT survey in terms of their ability to predict their probability to engage in high-risk trips. The top three individual predictors, number of children, lack of outside sleeping structure and age, would impact, on average, forest-goers’ probability to engage in high-risk trips by, 5%, 7% and 17% on the additive scale respectively and together defined strata of forest-goers with probability as high as 75%. In combination, these predictors separated the forest-going population into two subgroups with respective probabilities of engaging in such high-risk trips of 54% across the seven non-reference strata and 33% in the reference strata. This difference in risk could be leveraged to focus resources and efforts on the most high-risk forest-goers but the results also suggest that some level of risk is ubiquitous among forest-goers. In particular, this study failed to identify a very low-risk subgroup and malaria programmes across the GMS would miss some high-risk forest-goers if they were to further segment this population.

This study also demonstrated how GPS loggers can be used to measure fine-scale mobility patterns of rural and hard to access forest-going populations in the GMS. Thanks to PNs, we were able to recruit forest-goers in the study and train them on all aspects of the GPS loggers. Acceptability among forest-goers was high and this study proved its feasibility with very few data gaps thanks to the external charging device and additional batteries that were provided with the GPS loggers. GPS coordinates every 15 to 30 min along forest-going trips represent an incredibly rich dataset about forest-goers’ mobility patterns and interaction with their surrounding environment that could not be collected otherwise via surveys or mobile phone data.

However, data visualization highlighted that forest-goers did not carry the GPS loggers at all times, likely because the instructions insisted too much on the importance of carrying them during forest-going trips. As a result, the analysis was restricted to the 5% of GPS points that were collected during the 803 outdoor trips. This was a necessary step to ensure high-quality input data in our analyses but limits the effectiveness of using such GPS loggers. In addition, these data required substantial processing time and simple steps such as directly collecting the GPS coordinates of forest-goers’ house and the exact timing when the GPS logger was handed out would have significantly improved our experience.

This study has additional limitations. First, the definition of high-risk trips is subjective and based on a simplified version of the malaria ecosystem in the GMS where what matters is exposure to places with suspected higher exposure to mosquito vectors. Having prospective data on malaria infection among this cohort would be extremely valuable. Forest-goers recruited in FTAT were tested for malaria before being given GPS loggers, but reverse causality would have undermined the results from any association analysis and statistical power was low with only six forest-goers in the GPS component of the study testing positive for malaria cases by PCR (polymerase chain reaction). Additional information about the actual activities forest-goers engaged in during their outdoors trips would also have been valuable for a more comprehensive definition of risk. Second, the small sample size of 96 forest-goers in the regression analysis lacked sufficient variation in some individual level features to evaluate their association with high-risk trips. For instance, 95% of forest-goers who carried a GPS logger were male. Third and related, the forest-goers participating in the GPS logger component of the study were somewhat different from those that did not. This may be due to chance or bias in PNs’ recruitment of forest-goers or recruitment of PNs themselves. Ultimately, the quality of the data sample relies heavily of the aptitude of PNs to navigate their communities of forest-goers and recruit representative participants. As a consequence, the results may not generalize well to the whole 2,904 forest goers recruited in FTAT or even the 20,000 forest-goers or so estimated to reside in the study area^1^.

## Conclusion

In conclusion, this study illustrated how GPS loggers can be leveraged to measure and characterize fine-scale mobility patterns of forest-going populations in southern Lao PDR. The results highlighted the diversity within forest-going trips but could not clearly segment the role of forest-goers in malaria transmission in the GMS any further. These results shall be informative to malaria programmes across the region to tailor their interventions and messaging to high-risk populations and meet the objective of eliminating malaria by 2030 in the GMS [15, 16].

## Supplementary Information

**Additional file 1: S1.** GPS filtering algorithm.

**Additional file 2: Figure S2.** Dendogram from the hierarchical clustering algorithm. Starting from the bottom, every data point, i.e outdoor trip, is regrouped one a time into “leaves” (=cluster) until they are all in one big and uninformative cluster. The length of the “branches” quantifies the dissimilarity between the leaves. The red horizontal line represents our subjective decision to cut the tree in 6 clusters. We felt selecting 5 clusters would have failed to cut lengthy branches whereas selecting 7 clusters would have started to cut short branches.

## Data Availability

The datasets used and/or analysed during the current study are available from the corresponding author on reasonable request.
