# Supplementary material for: Characterizing mobility patterns of forest goers in southern Lao PDR using GPS loggers
Source: Malar J. 2023 Feb 2;22:38. doi: 10.1186/s12936-023-04468-8 (PMC9893532; doi:10.1186/s12936-023-04468-8)
Supplement: Supplementary file 1 — Additional file 1: S1. GPS filtering algorithm. [file 12936_2023_4468_MOESM1_ESM.docx]

Characterizing mobility patterns of forest goers

in southern Lao PDR using GPS loggers.

Author Block: Francois Rerolle^1,2*^, Emily Dantzer^1^, Toula Phimmakong^3^, Andrew Lover^4^, Bouasy Hongvanthong^3^, Rattanaxay Phetsouvanh^5^, John Marshall^6^, Hugh Sturrock^1,2^, Adam Bennett^1,2^

^1^Malaria Elimination Initiative, The Global Health Group, University of California, San Francisco, CA, USA, ^2^Department of Epidemiology and Biostatistics, University of California, San Francisco, CA, USA, ^3^Center for Malariology, Parasitology and Entomology, Ministry of Health, Vientiane, Lao People's Democratic Republic, ^4^Department of Biostatistics and Epidemiology, School of Public Health and Health Sciences, University of Massachusetts-Amherst, MA, USA, ^5^Department of Communicable Disease Control, Ministry of Health, Vientiane, Lao PDR, ^6^Divisions of Epidemiology and Biostatistics, School of Public Health, University of California, Berkeley, CA, USA

*For correspondence: francois.rerolle@ucsf.edu

**Additional file 1: S1: GPS filtering algorithm**

The advertised precision of the I-gotU GPS loggers used in this study is 10m. Yet, the constructor warns of possible large errors in the GPS coordinates collected, notably when the logger stayed indoor for long periods of time and cannot connect with the satellites. To remove those erroneous GPS points, we used a filtering algorithm that identifies GPS points unusually far away from both the previous and next GPS points, themselves being close by.

Our filtering algorithm first identifies suspect GPS points when the average speed leading to such point from the previously logged one is above 3 km/h (and time difference > 1 min). These suspect points essentially look like suddenly “motorized” departures. Second, the filtering algorithm groups with the identified suspect point, all subsequent points that were recorded really quickly (time difference < 1 min) afterwards. This is because our standard operating procedures documents failed to stress enough that the data logging frequency should not depend on the detected speed of the device. See methods. Third, time difference, distance and elevation between the GPS points starting and ending the sequence of suspect points were computed. Last, the sequence of suspect GPS points (often times comprising only 1 data point) were filtered out according to the following decision rules:

- (Distance between starting and ending suspect GPS points < 100 m) AND (Time between starting and ending suspect GPS points < 30 min)

OR

- (Distance between starting and ending suspect GPS points < 100 m) AND (Time between starting and ending suspect GPS points < 60 min) AND ((Elevation difference with previous GPS point > 500 ft) OR (Average speed between previous and current GPS point > 10 km/h))

OR

- (Distance between starting and ending suspect GPS points < 100 m) AND (Time between starting and ending suspect GPS points < 90 min) AND ((Elevation difference with previous GPS point > 1000 ft) OR (Average speed between previous and current GPS point > 25 km/h))

OR

- (Distance between starting and ending suspect GPS points < 3000 m) AND (Time between starting and ending suspect GPS points < 180 min) AND ((Elevation difference with previous GPS point > 3000 ft) OR (Average speed between previous and current GPS point > 40 km/h))

The decision rules were designed to filter out unusual sequence of GPS points between otherwise very close GPS points (before and after). Bumps in elevation difference were also indicative of a temporary dysfunction in the GPS logger and leveraged as such. Unusual speed, even considering the possibility of motor transportation were also used to identify erroneous GPS points. Importantly, these decision rules were refined in an iterative process by visually inspecting the effect of the filtering algorithm on the GPS trajectories.

In addition, our standard operating procedures documents about the configurations of the GPS loggers failed to stress enough that we did not want the data logging frequency to depend on the detected speed of the device. As a result, for some GPS loggers correctly logging most of their GPS coordinates every 15 to 30 min, the device, when detecting high speed, switched to "motorized" mode and collected GPS coordinates every second. When that happened, we trimmed the data to keep a GPS points every 3 min in order to reduce the computational time in data processing methods described below.
