# Supplementary material for: Characterizing mobility patterns of forest goers in southern Lao PDR using GPS loggers
Source: Malar J. 2023 Feb 2;22:38. doi: 10.1186/s12936-023-04468-8 (PMC9893532; doi:10.1186/s12936-023-04468-8)
Supplement: Supplementary file 2 — Additional file 2: Figure S2. Dendogram from the hierarchical clustering algorithm. Starting from the bottom, every data point, i.e outdoor trip, is regrouped one a time into “leaves” (=cluster) until they are all in one big and uninformative cluster. The length of the “branches” quantifies the dissimilarity between the leaves. The red horizontal line represents our subjective decision to cut the tree in 6 clusters. We felt selecting 5 clusters would have failed to cut lengthy branches whereas selecting 7 clusters would have started to cut short branches. [file 12936_2023_4468_MOESM2_ESM.docx]

Characterizing mobility patterns of forest goers

in southern Lao PDR using GPS loggers.

Author Block: Francois Rerolle^1,2*^, Emily Dantzer^1^, Toula Phimmakong^3^, Andrew Lover^4^, Bouasy Hongvanthong^3^, Rattanaxay Phetsouvanh^5^, John Marshall^6^, Hugh Sturrock^1,2^, Adam Bennett^1,2^

^1^Malaria Elimination Initiative, The Global Health Group, University of California, San Francisco, CA, USA, ^2^Department of Epidemiology and Biostatistics, University of California, San Francisco, CA, USA, ^3^Center for Malariology, Parasitology and Entomology, Ministry of Health, Vientiane, Lao People's Democratic Republic, ^4^Department of Biostatistics and Epidemiology, School of Public Health and Health Sciences, University of Massachusetts-Amherst, MA, USA, ^5^Department of Communicable Disease Control, Ministry of Health, Vientiane, Lao PDR, ^6^Divisions of Epidemiology and Biostatistics, School of Public Health, University of California, Berkeley, CA, USA

*For correspondence: francois.rerolle@ucsf.edu

**Additional file 2: S2: Additional figures**


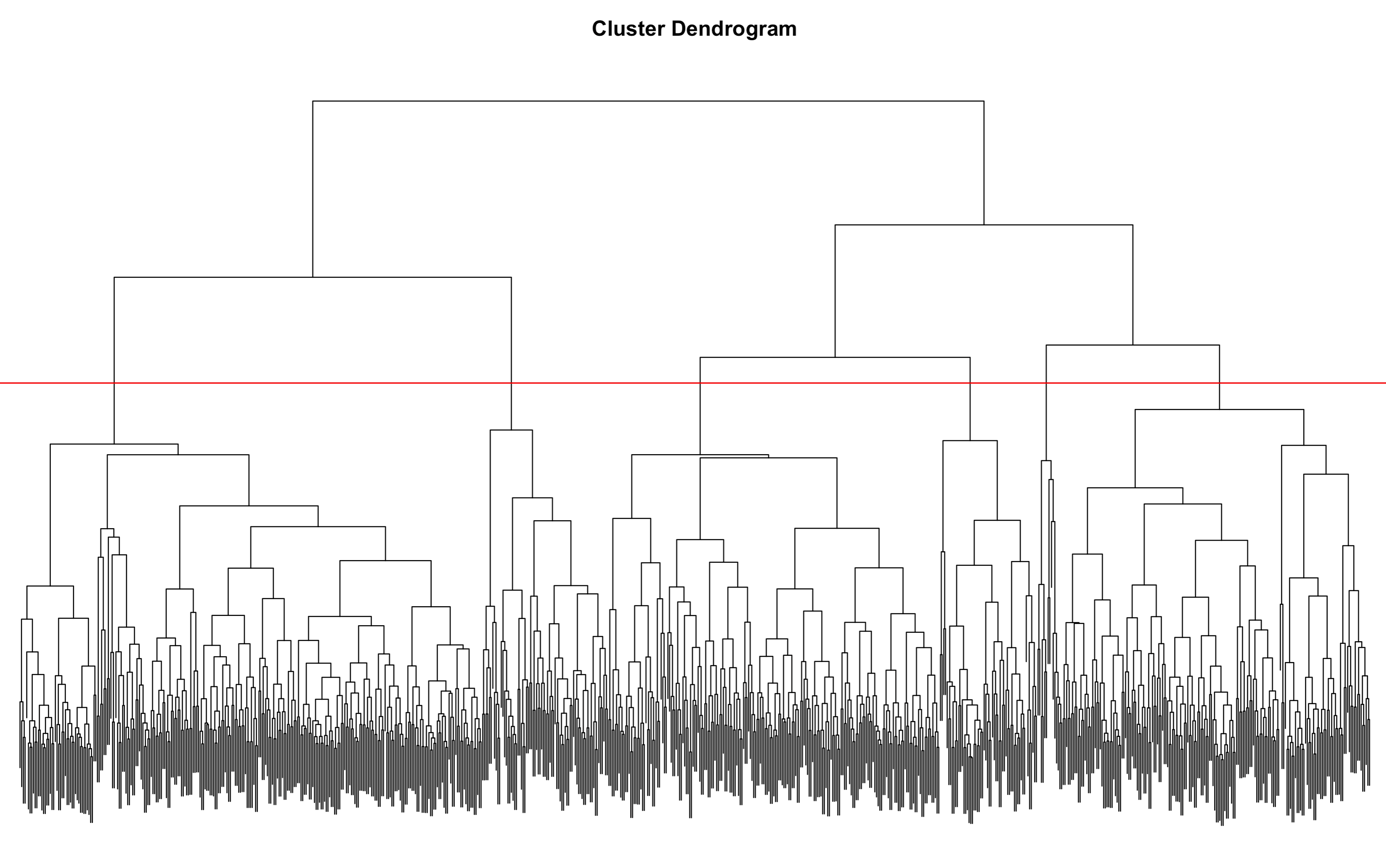


**Figure S2.1 –** *Dendogram from the hierarchical clustering algorithm. Starting from the bottom, every data point, i.e outdoor trip, is regrouped one a time into “leaves” (=cluster) until they are all in one big and uninformative cluster. The length of the “branches” quantifies the dissimilarity between the leaves. The red horizontal line represents our subjective decision to cut the tree in 6 clusters. We felt selecting 5 clusters would have failed to cut lengthy branches whereas selecting 7 clusters would have started to cut short branches.*
